# Molecular Mechanism of Aerobic Exercise Ameliorating Myocardial Mitochondrial Injury in Mice with Heart Failure

**DOI:** 10.3390/ijms26052136

**Published:** 2025-02-27

**Authors:** Hao Jia, Yinping Song, Yijie Hua, Kunzhe Li, Sujuan Li, Youhua Wang

**Affiliations:** Institute of Sports and Exercise Biology, School of Physical Education, Shanxi Normal University, Xi’an 710119, China; 19710125504@163.com (H.J.); syp@xafy.edu.cn (Y.S.); 22211010002@m.fudan.edu.cn (Y.H.); mm1321751045@163.com (K.L.); 18768960912@163.com (S.L.)

**Keywords:** oxidative stress, aerobic exercise, heart failure, mitochondrial quality control, inflammation, apoptosis

## Abstract

To explore the molecular mechanism of aerobic exercise to improve heart failure and to provide a theoretical basis and experimental reference for the treatment of heart failure. Nine-week-old male mice were used to establish a left ventricular pressure overload-induced heart failure model by transverse aortic constriction (TAC). The mice were randomly divided into four groups: a sham group (SHAM), heart failure group (HF), heart failure + SKQ1 group (HS) and heart failure + aerobic exercise group (HE). The mice in the HE group were subjected to moderate-intensity aerobic exercise interventions. The mitochondrion-targeting antioxidant (SKQ1) contains the lipophilic cation TPP, which targets scavenging mitochondrial ROS. The HS group was subjected to SKQ1 (100 nmol/kg/d) interventions, which were initiated 1 week after the surgery, and the interventions lasted 8 weeks. Cardiac function was assessed by ultrasound, cardiomyocyte size by H&E and WGA staining, myocardial fibrosis by Masson’s staining, and myocardial tissue oxidative stress and apoptosis by DHE and TUNEL fluorescence staining, respectively. Western blotting was used to detect the expression of mitochondrial quality control, inflammation, and apoptosis-related proteins. In the cellular level, an in vitro cellular model was established by isolating primary cardiomyocytes from neonatal mice (2–3 days) and intervening with Ang II (1 μM) to mimic heart failure. Oxidative stress and mitochondrial membrane potential were determined in the cardiomyocytes of each group by DHE and JC-1 staining, respectively. Myocardial fibrosis was increased significantly and cardiac function was reduced significantly in the heart failure mice. Aerobic exercise and SKQ1 intervention improved cardiac function and reduced myocardial hypertrophy and myocardial fibrosis in the heart failure mice significantly. Meanwhile, aerobic exercise and SKQ1 intervention reduced the number of DHE-positive particles (*p* < 0.01) and inhibited myocardial oxidative stress in the heart failure mice significantly. Aerobic exercise also reduced DRP1, Parkin, and BNIP3 protein expression (*p* < 0.05, *p* < 0.01), and increased OPA1 and PINK1 protein expression (*p* < 0.05, *p* < 0.01) significantly. Moreover, aerobic exercise and SKQ1 intervention decreased the number of TUNEL-positive particles and the expression of inflammation- and apoptosis-related proteins NLRP3, TXNIP, Caspase-1, IL-1β, BAX, BAK, and p53 significantly (*p* < 0.05, *p* < 0.01). In addition, the AMPK agonist AICAR and the mitochondria-targeted ROS scavenger (SKQ1) ameliorated AngII-induced mitochondrial fragmentation and decreased mitochondrial membrane potential in cardiomyocytes significantly. It was shown that inhibition of mitochondrial ROS by aerobic exercise, which in turn inhibits mitochondrial damage, improves mitochondrial quality control, and reduces myocardial inflammatory and apoptosis, may be an important molecular mechanism by which aerobic exercise exerts endogenous antioxidant protective effects to improve cardiac function.

## 1. Introduction

Heart failure (HF) is an exponentially growing epidemiological syndrome with a prevalence of up to 10 percent in people aged 70 years and older and a global prevalence of approximately 37.7 million people [1,2]. Accumulation of ROS pressure is particularly common in high-frequency heart failure due to the high energy demands of cardiomyocytes and the dense distribution of mitochondria [3]. Excess ROS can trigger the opening of the mitochondrial permeability conversion pore and cause severe inflammatory response and myocardial apoptosis, leading to heart failure [4,5]. There is substantial evidence that the use of antioxidants in elderly or chronically ill patients helps to reduce the incidence of cardiovascular events [6]. However, antioxidant therapies such as vitamin C are not effective in clinical management [7]. The mitochondrion-targeting antioxidant SKQ1 (Visomitin) contains the lipophilic cation TPP, which targets scavenging mitochondrial ROS. Studies have found that SKQ1 can reduce lipid peroxidation level, reduce the number of neutrophils, inhibit inflammatory response, and then alleviate cardiac hypertrophy [8]. Other studies confirmed that 8 weeks of exercise training increased myocardial antioxidant capacity and decreased myocardial malondialdehyde levels in rats, as well as reduced cardiac damage from oxidative stress [9]. In addition, aerobic exercise can improve the exercise ability and quality of life of patients with heart failure, and has a protective effect on heart failure complications [10,11,12,13]. However, the specific mechanism of aerobic exercise to improve heart failure is still unclear, especially against endogenous targets.

Aerobic exercise ameliorates cardiovascular disease by mobilizing multiple signaling pathways involved in various biological processes within cardiomyocytes, such as muscle-secreted irisin and liver-secreted FGF21 [14]. Regular exercise was found to increase the expression of major antioxidant enzymes and decrease pro-oxidant enzymes [15]. In addition, exercise activates an antioxidant response in diabetic hearts through the AMPK/PGC-1α axis, enhances membrane potential, and reduces ROS levels [16]. However, resistance training up-regulates FGF21 protein expression, inhibits activation of the TGF-β1-Smad2/3-MMP2/9 signaling pathway and collagen production, enhances cardiac antioxidant capacity, and reduces apoptosis in myocardial infarction mice [14]. However, the molecular mechanism by which aerobic exercise improves heart failure by activating endogenous antioxidants has been largely unknown. Therefore, this study focused on the effects of aerobic exercise and SKQ1 inhibition of mitochondrial ROS on myocardial oxidative stress, mitochondrial damage, myocardial inflammation, and apoptosis in mice with heart failure, and explained the molecular mechanism of aerobic exercise inhibition of mitochondrial ROS to improve heart failure. It also explains the molecular mechanism of mitochondrial ROS inhibition by aerobic exercise to improve heart failure.

## 2. Results

### 2.1. Aerobic Exercise Significantly Reduces TAC-Induced Cardiac Dysfunction

**Figure 1:**
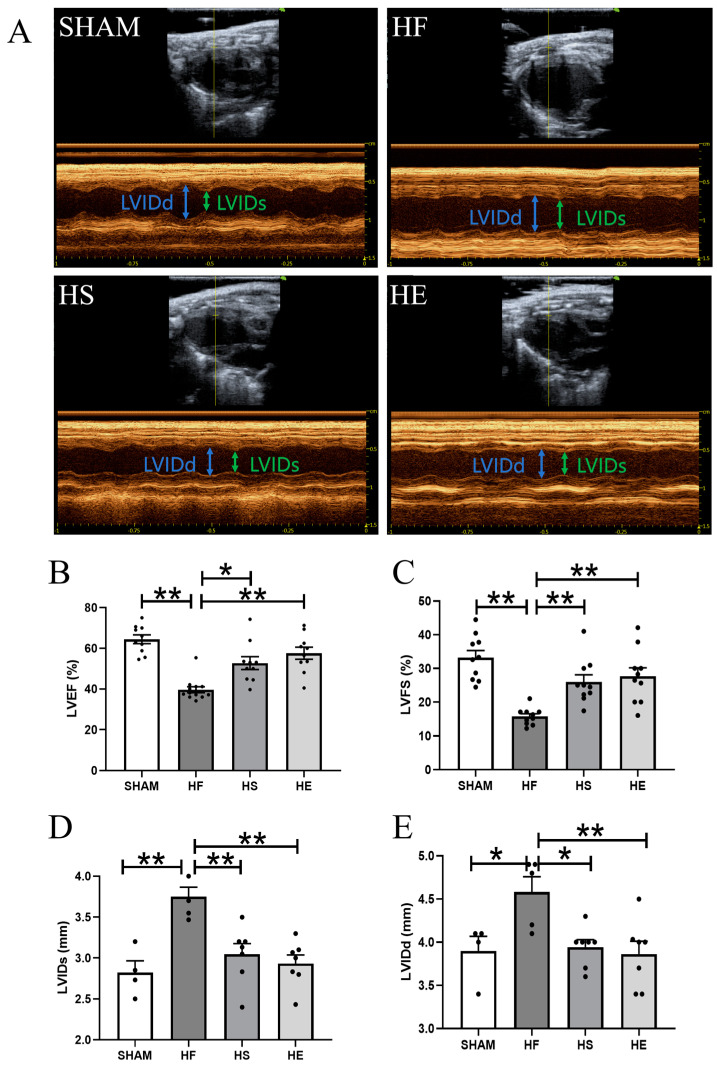
Aerobic exercise reduces TAC-induced cardiac dysfunction. (**A**) Representative images of cardiac ultrasound in each group; (**B**–**E**) were the quantitative analysis of left ventricular ejection fraction (LVEF), left ventricular short-axis shortening rate (LVFS), left ventricular internal diameter at end-systole (LVIDs), and left ventricular end-diastolic internal diameter (LVIDd) in each group. * indicates *p* < 0.05 compared with the HF group, ** indicates *p* < 0.01 compared with the HF group.

Cardiac ultrasound was used to evaluate cardiac function in each group of mice. The heart rate of the HF and HE groups was slightly higher, but there was no significant difference between the groups, so the influence of anesthesia on the determination of cardiac function was excluded. In the heart failure group, the left ventricular end-diastolic internal diameter and left ventricular internal diameter at the end-systole were significantly increased, the left ventricular cavity was enlarged, the ejection fraction (EF) and brachyaxis shortening rate (FS) were significantly decreased, and the EF < 40% and FS < 20%, indicating that the heart failure model was successfully constructed. Aerobic exercise and SKQ1 significantly reduced TAC-induced cardiac dysfunction (Figure 1A–E).

### 2.2. Aerobic Exercise Significantly Reduces TAC-Induced Myocardial Remodeling

**Figure 2:**
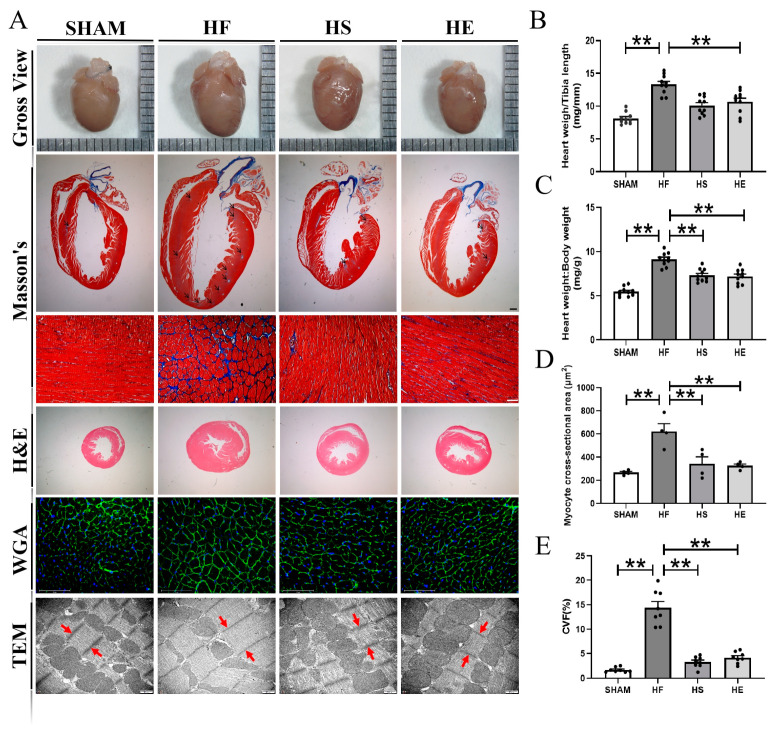
Aerobic exercise reduces TAC-induced cardiomyocyte hypertrophy and myocardial fibrosis. (**A**) Representative images of the heart as a whole, Masson’s, H&E, and WGA staining for each group, respectively; scale bars are 1 mm, 50 μm, and 75 μm; myocardial sarcomere length was assessed by transmission electron microscopy, with red arrows pointing to the Z line, and two red arrows denoting the sarcomere lengths; (**B**) cardiac–tibial ratio; (**C**) cardiac–body ratio; (**D**) quantitative analysis of the cross-sectional area of cardiomyocytes for each group; (**E**) myocardial quantitative analysis of fibrosis (CVF). ** indicates *p* < 0.01 compared with the HF group.

In order to evaluate the degree of myocardial hypertrophy, the heart weight, body weight, and tibia length were measured, respectively. The hearts of the mice in the heart failure group were significantly enlarged (Figure 2A), and the heart-to-shin ratio and heart-to-body ratio were significantly increased. Aerobic exercise and SKQ1 intervention significantly reduced the heart-to-shin ratio and heart-to-body ratio (Figure 2B,C). In addition, by H&E, WGA, and Masson’s staining, it was found that the cardiomyocytes of mice in the heart failure group were significantly hypertrophic and the area of myocardial collagen was significantly increased. Aerobic exercise and SKQ1 significantly reduced TAC-induced myocardial remodeling (Figure 2D,E).

### 2.3. Aerobic Exercise and SKQ1 Inhibit Myocardial Oxidative Stress Levels

**Figure 3:**
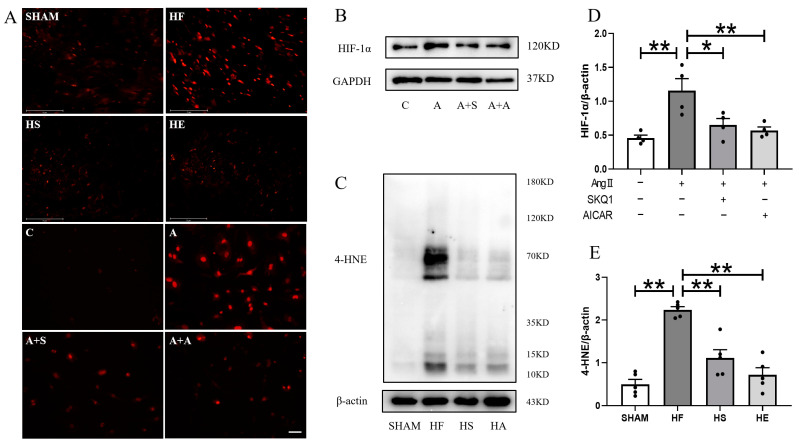
Aerobic exercise and SKQ1 reduce myocardial oxidative stress. (**A**) Representative images of DHE (red) staining of cardiac tissues in each group with a scale bar of 75 μm, and representative images of DHE staining of primary cardiomyocytes in each group with a scale bar of 50 μm. (**B**) Expression of HIF-1α in cardiomyocytes in each group. (**C**) Expression of 4-HNE in cardiomyocytes in each group. (**D**) Images of quantitative analysis of HIF-1α in cardiac tissues in each group. (**E**) Images of 4-HNE in cardiac tissues in each group. quantitative analysis images. * indicates *p* < 0.05 compared with the HF group, ** indicates *p* < 0.01 compared with the HF group.

In order to evaluate the effect of aerobic exercise and SKQ1 on inhibiting oxidative stress, DHE staining was determined. The positive particles and the expression of 4-HNE in the cardiac tissue of mice in the heart failure group were significantly increased. Aerobic exercise and SKQ1 significantly inhibited myocardial oxidative stress and lipid peroxidation levels in mice with heart failure (Figure 3A,C,E). Similarly, the DHE signal and HIF-1α expression in myocytes induced by Ang II were significantly enhanced. AICAR and SKQ1 intervention reduced the level of oxidative stress in cardiomyocytes (Figure 3A,D).

### 2.4. Aerobic Exercise Inhibits Oxidative Stress-Mediated Mitochondrial Damage and Improves Mitochondrial Quality Control

**Figure 4:**
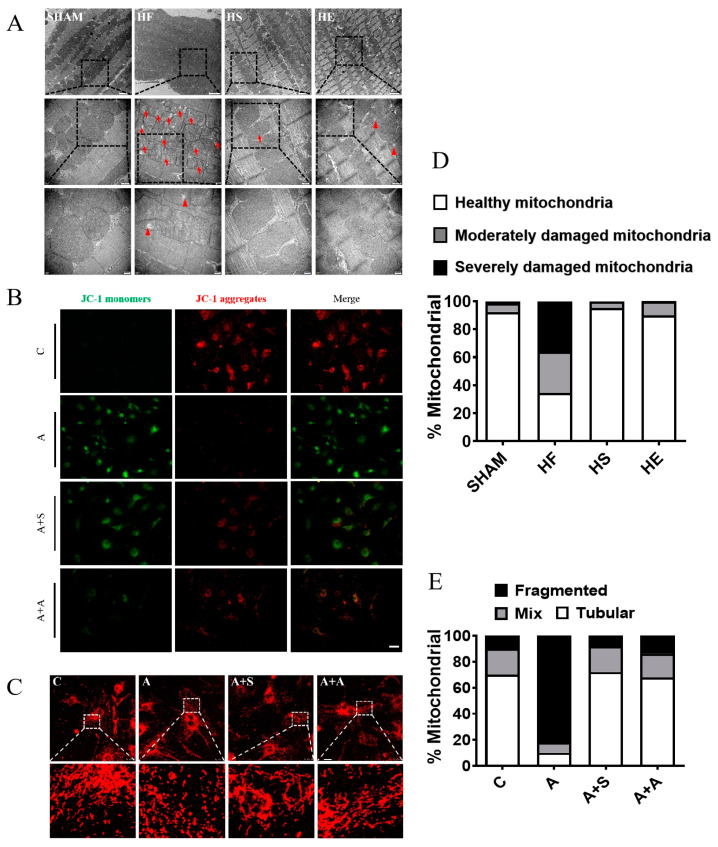
Aerobic exercise and SKQ1 improve myocardial mitochondrial damage. (**A**) TEM showing the ultrastructure of myocardial mitochondria in each group. Red arrows point to cristobaltic mitochondria; red triangles point to mitochondrial vacuolization; the images were enlarged step by step, with scale bars of 2 μm, 500 nm, and 200 nm, respectively. (**B**) Cardiomyocytes of each group were stained with MitoTracker Red (red), and short, rounded mitochondria indicate fragmentation, with a scale bar of 25 μm. (**C**) JC-1 staining of cardiomyocytes in each group, producing red fluorescence when the mitochondrial membrane potential was high and green fluorescence when the mitochondrial membrane potential was low, with a scale bar of 100 μm. (**D**) Percentage of damaged mitochondria was counted according to TEM. (**E**) Statistical graph of mitochondrial fragmentation.

To evaluate myocardial mitochondrial damage, the ultrastructure was observed by transmission electron microscopy. In the heart failure group, the myocardial fibers were disordered, the myotome was longer, the transverse lines were blurred, the mitochondrial swelling and cavitation were obvious, the membrane structure was damaged, the mitochondrial ridge was dissolved, and the autophagosomes were obvious. Aerobic exercise and SKQ1 inhibited oxidative stress-mediated mitochondrial damage (Figure 4A). Further in vitro experiments using Mito Tracker Red staining showed AngII-induced mitochondrial fragmentation of cardiomyocytes. The number of mitochondria in the cytoplasm of Ang II group increased, the length of mitochondria was shortened, the shape of mitochondria was similar to a ball, and the mitochondrial fragmentation was serious. The intervention of AICAR and SKQ1 increased mitochondrial length, significantly reduced the number of globular mitochondria, and significantly improved mitochondrial fragmentation (Figure 4B). JC-1 staining was used to evaluate mitochondrial membrane potential. In the control group, the green fluorescence was weak, the red fluorescence was strong, and the mitochondrial membrane potential was high. In the Ang II group, the red fluorescence was decreased, the green fluorescence was increased, and the mitochondrial membrane potential induced by Ang II was decreased. SKQ1 and AICAR increased the mitochondrial membrane potential, and the effect of AICAR was more obvious (Figure 4C).

**Figure 5:**
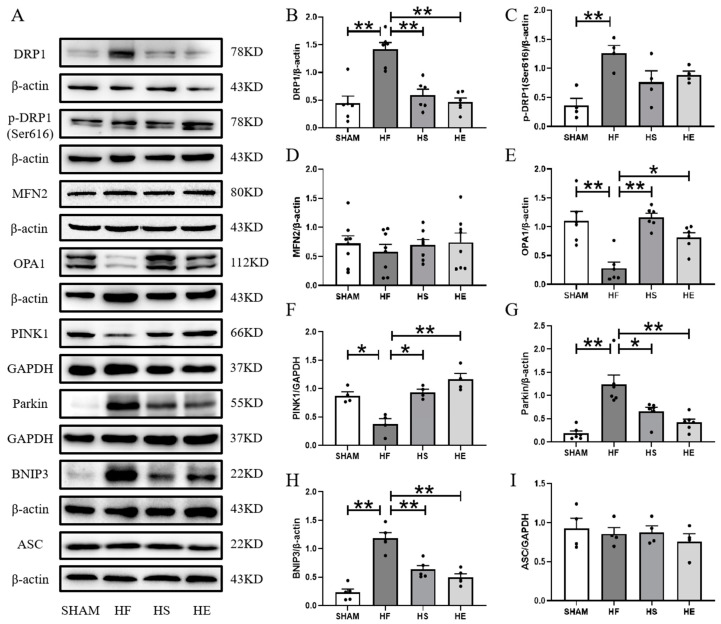
Inhibition of mitochondrial ROS by aerobic exercise and SKQ1 intervention regulates mitochondrial division, fusion, and mitochondrial autophagy. (**A**–**I**) Expression representative images and quantitative statistics of DRP1, p-DRP1 (Ser616), OPA1, MFN2, PINK1, Parkin, BNIP3, and ASC in myocardial tissue. * indicates *p* < 0.05 compared with HF group, ** indicates *p* < 0.01 compared with HF group.

Mitochondrial dynamics disorder plays a crucial role in the pathogenesis of heart failure [17,18,19]. Western blotting results showed that the expressions of MFN2 and OPA1 proteins were down-regulated in the heart failure group, and up-regulated by aerobic exercise and SKQ1 (Figure 5D,E). DRP1 is a GTP enzyme with multiple phosphorylation sites, among which serine phosphorylation at site 616 promotes its recruitment to mitochondria, which is necessary for mitochondrial fission [17]. The expression of DRP1 protein and phosphorylation of DRP1 Ser616 site were significantly up-regulated in the heart failure group. Aerobic exercise and SKQ1 significantly down-regulated DRP1 protein expression and down-regulated serine phosphorylation at site 616 (Figure 5B,C). Down-regulation of mitochondrial autophagy plays an important role in mediating the occurrence and development of mitochondrial dysfunction and heart failure [20]. In the heart failure group, PINK1 protein expression was significantly down-regulated, Parkin and BNIP3 protein expression were significantly up-regulated, and SKQ1 and aerobic exercise significantly down-regulated Parkin and BNIP3 protein expression (Figure 5F–H).

### 2.5. Aerobic Exercise Reduces Oxidative Stress-Mediated Myocardial Inflammation and Apoptosis

**Figure 6:**
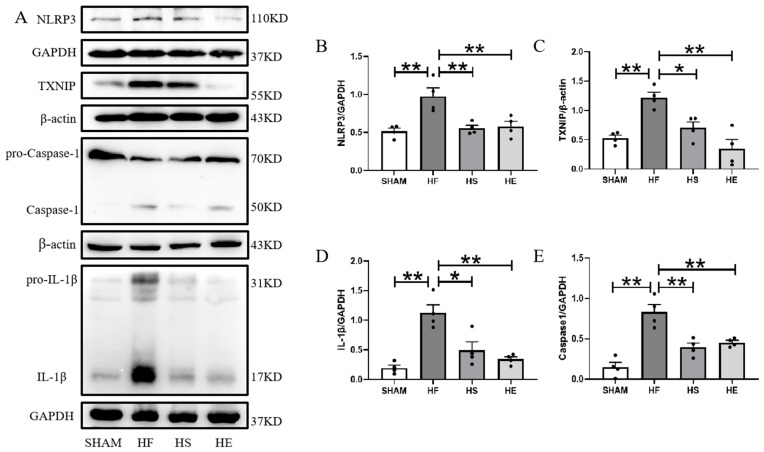
Inhibition of mitochondrial ROS by aerobic exercise and SKQ1 intervention reduces myocardial inflammation. (**A**–**E**) Representative images and quantitative statistical plots of myocardial tissue expression of inflammation-related proteins NLRP3, TXNIP, Caspase-1, and IL-1β. * indicates *p* < 0.05 compared with HF group, ** indicates *p* < 0.01 compared with HF group.

Inflammasome is a key mediator of cardiac inflammation during heart failure. Activation of the NLRP3 inflammasome has been shown to induce myocardial hypertrophy under pressure overload [21]. In the heart failure group, NLRP3, TXNIP, Caspase-1, and IL-1β protein expressions were up-regulated, and aerobic exercise and SKQ1 down-regulated NLRP3, TXNIP, Caspase-1, and IL-1β protein expressions (Figure 6A–E).

**Figure 7:**
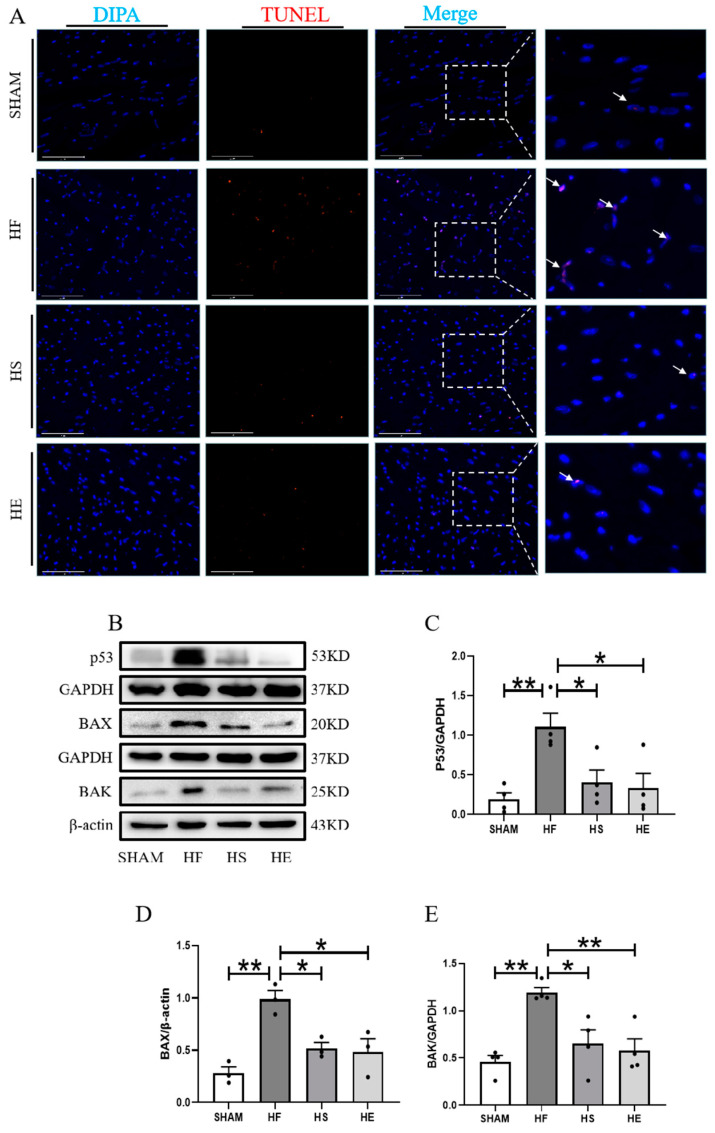
Inhibition of mitochondrial ROS by aerobic exercise and SKQ1 intervention reduces myocardial apoptosis. (**A**) Cardiac tissues of each group were stained with TUNEL (red) and DAPI (blue), respectively; white arrows indicate TUNEL-positive particles, with a scale bar of 75 μm. (**B**–**E**) Representative images and quantitative statistic graphs of apoptosis-related proteins p53, BAK, and BAX expression in myocardial tissues. * indicates *p* < 0.05 compared with the HF group, ** indicates *p* < 0.01 compared with the HF group.

Apoptosis is a unique form of cell death, and myocardial cell apoptosis is a key mechanism in the pathogenesis of heart failure [22]. Western blotting and TUNEL detection showed that TUNEL-positive particles increased significantly, and the protein expressions of p53, BAX, and BAK were significantly up-regulated in the heart failure group. Aerobic exercise and SKQ1 decreased the number of TUNEL positive nuclei and down-regulated the expression of p53, BAX, and BAK proteins (Figure 7A–E).

## 3. Discussion

### 3.1. Molecular Mechanisms of Targeted Scavenging of Mitochondrial ROS to Inhibit Myocardial Injury

The pathogenesis of heart failure is still unclear, but the more widely accepted theory is that heart failure is caused by cardiac oxidative stress and inflammation, accompanied by apoptosis and myocardial injury [23,24]. Enhanced mitochondrial ROS release and increased mitochondrial damage have been found in failing hearts [18,25]. The cytoplasmic and mitochondrial ROS levels of isolated failing cardiomyocytes were significantly increased [21]. Our experimental results were similar. Myocardial and Ang II-induced oxidative stress of cardiomyocytes in mice with heart failure significantly increased, mitochondrial membrane potential decreased, and fragmentation intensified. Therefore, inhibition of oxidative stress is a key strategy in the treatment of heart failure, and the search for cardiovascular antioxidants has never ceased; however, large clinical trials have not shown significant efficacy of antioxidants (e.g., vitamin C) in cardiovascular disease [22,26]. One important explanation for this is that substances such as vitamin C do not target the most important sites of ROS generation under pathological conditions, such as mitochondria, which are responsible for more than 90% of the ROS in the cell [22].

SKQ1 contains the lipophilic cation TPP, and the positive charge of TPP+ enables it to quickly pass through the phospholipid bilayer and accumulate in the mitochondrial matrix through electrochemical gradient drive, thus targeting the removal of mitochondrial ROS [23,26,27]. Studies have confirmed that a long-term high-fructose diet can lead to heart enlargement and diastolic dysfunction as well as elevated mitochondrial ROS. However, administration of SKQ1 inhibited mitochondrial ROS and prevented cardiac hypertrophy [24]. Other studies have found that SkQ1 protects myocardial mitochondria by reducing mitochondrial ROS content, improves the ultrastructure of myocardial mitochondria in rats, and alleviates mitochondrial damage [28]. Consistent with our observation, SKQ1 can protect the structural integrity of myocardial mitochondria, inhibit mitochondrial fragmentation of cardiomyocytes, increase membrane potential, and weaken mitochondrial damage. Cardiolipin is a mitochondrial phospholipid that is particularly sensitive to ROS attacks. SkQ1 can prevent the oxidation of cardiolipin and prevent the vicious cycle of cardiolipin remodeling, lipid peroxidation, and mitochondrial dysfunction induced by cardiolipin oxidation [29,30,31]. Studies have shown that SKQ1 can inhibit mitochondrial ROS, reduce lipid peroxidation levels [32], inhibit cardiolipin oxidation, stabilize the mitochondrial electron transport chain, and significantly attenuates adriamycin-induced cardiomyocyte injury [26]. SkQ1 also inhibited mitochondrial ROS, reduces mtDNA oxidation, and then inhibits the expression of NLRP3, Caspase-1, and IL-1β, thus blocking ox-mtDNA from binding to the mitochondria. SkQ1 also showed strong anti-inflammatory effects by inhibiting mitochondrial ROS, reducing mtDNA oxidation and thereby inhibiting the expression of NLRP3, Caspase-1, and IL-1β, thus blocking the interaction between ox-mtDNA and NLRP3. This in turn inhibits NLRP3 inflammasome activation and NLRP3-mediated inflammatory signaling [28,33]. In addition, SKQ1 inhibited leukocyte and mesangial cell adhesion and/or infiltration by reducing the expression of adhesion proteins, thereby reducing inflammation and subsequent diffuse myocardial fibrosis [34,35,36]. Similarly to our results, the NLRP3/caspase-1/IL-1β signaling pathway was inhibited by SKQ1 intervention to inhibit mitochondrial ROS, Reduce the expression of BAX, Bak, p53 proteins, inhibit cell apoptosis, and ultimately improve myocardial fibrosis, relieve myocardial damage, and protect cardiac function. Therefore, inhibition of mitochondrial ROS is a key step in the treatment of heart failure.

### 3.2. Molecular Mechanisms by Which Aerobic Exercise Inhibits Oxidative Stress-Induced Mitochondrial Damage and Improves Mitochondrial Quality Control

The eruption of mitochondrial ROS and the ensuing oxidative stress can trigger the opening of the mitochondrial permeability transition pore, affect mitochondrial dynamics and mitochondrial autophagy, and induce mitochondrial damage [17,18,37]. Current studies have shown that mitochondrial fusion is inhibited and mitochondrial division is promoted in heart failure [19,38,39]. Mitochondrial division inhibitor-1 can inhibit myocardial hypertrophy induced by pressure overload and reduce cardiomyocyte hypertrophy induced by exercise training via reducing DRP1 levels [40]. In addition, exercise increases the activities of MFN1 and MFN2GTPase in myocardium, reverses the translocation of DRP1 to mitochondria, promotes mitochondrial dynamic remodeling, and effectively alleviates mitochondrial dysfunction in rats with myocardial infarction [41,42,43]. Similar results were obtained in this study, which showed that aerobic exercise could regulate mitochondrial dynamics, induce increased mitochondrial fusion, decreased mitochondrial fission, and reduced mitochondrial fragmentation.

Mitochondrial autophagy can remove damaged mitochondria from the heart muscle. The current research on mitochondrial autophagy in cardiovascular diseases mainly focuses on the PINK1/parkin pathway. Knockout of the PINK1 gene leads to an increased accumulation of damaged mitochondria, resulting in a pathologic imbalance in ROS generation and leading to mitochondrial bioenergetics disruption [44]. Insufficient or excessive mitochondrial autophagy can aggravate the development of heart failure. TAC leads to dynamic changes in myocardial autophagy and mitochondrial autophagy. In the early stage of HF, mitochondrial autophagy in heart tissue increases briefly, but it is down-regulated in the chronic stage of TAC-induced HF [45]. However, our previous study observed that BNIP3 protein showed a trend of decreasing first and then increasing, and the expression was most significant at the eighth week of TAC. Meanwhile, Parkin protein expression continued to be up-regulated and began to be slightly down-regulated and maintained at a high level after peaking at week 5 [46]. In the present study, the expression of PINK1 protein was significantly decreased and Parkin expression was significantly increased in the myocardium of heart failure mice. MFN2 is a substrate for the ubiquitination of Parkin and PINK1 phosphorylates and promotes Parkin-mediated ubiquitination of MFN2 and Parkin translocation to the damaged mitochondria [47]. In this study, although the expression of Parkin was increased in mice with heart failure, MFN2 expression did not change significantly, suggesting that the mitochondrial translocation of Parkin was reduced and mitochondrial autophagy was inhibited [20,45]. Aerobic exercise training enhances the PINK1/Parkin signaling pathway, thereby inducing mitochondrial autophagy [48]. Furthermore, it can enhance the antioxidant capacity of the body, improve the quality of mitochondria, and help alleviate the cardiac dysfunction of mice after myocardial infarction [41,48]. We intervened with SKQ1 in our experiments and found that aerobic exercise had a similar ameliorative effect with SKQ1. Therefore, we hypothesized that aerobic exercise could inhibit mitochondrial ROS, control ROS within physiological levels, reduce mitochondrial damage, and then improve mitochondrial division, fusion, and autophagy, and promote the health of mitochondrial dynamic network.

### 3.3. Aerobic Exercise Activates Endogenous Antioxidants to Alleviate Mitochondrial Damage-Induced Myocardial Inflammation and Apoptosis

Oxidative stress and mitochondrial damage form a vicious cycle to induce myocardial inflammation and apoptosis, which aggravate the occurrence and development of heart failure [49]. When mitochondria produce excessive ROS, TXNIP is induced to shuttle into the cytoplasm or mitochondria, inhibiting the antioxidant capacity of Trx by binding to it and further increasing ROS accumulation [50]. Endoplasmic reticulum stress and mitochondrial stress are induced, while TXNIP activates NLRP3 protein, which binds to Caspase-1 and ASC, and the assembled NLRP3 inflammasome can activate the protease Caspase-1 and promote the release of IL-1β [49,51]. In the presence of sustained ROS production, mitochondrial damage is exacerbated and the intrinsic pathway of apoptosis is triggered [52]. When apoptosis is triggered, the retrograde translocation of BAX to cytosol is stopped, and two pro-apoptotic proteins, BAK and BAX, are activated by factors of pro-apoptotic BH3 [53]. BAX and BAK insert into the outer mitochondrial membrane and oligomerize, forming a transmembrane pore that allows apoptotic factors to flow out [54]. The assembly of such pore structures increases membrane permeability in mitochondria, further aggravates mitochondrial damage, and worsens heart failure [55]. Studies have shown that inhibiting NLRP3 is a feasible strategy to reduce adverse cardiac remodeling and improve left ventricular function in patients with heart failure, and blocking the NLRP3 inflammatory pathway has potential benefits for patients with heart failure [56]. Ferulic acid ameliorated isoproterenol-induced heart failure in rats by reducing oxidative stress and inhibiting cardiomyocyte apoptosis through the activation of the NRF2 pathway [57]. Other studies found that TAC-induced myocardial hypertrophy can induce increased myocardial BAK expression, and the increase in BAK can lead to the escape of mitochondrial DNA (mtDNA), resulting in a decrease in the number of mtDNA copies, which further worsens heart failure [54]. This study found that aerobic exercise and SKQ1 can inhibit the mtDNA escape effect by inhibiting the production of ROS, and then inhibiting the increase in BAK expression, thereby preventing the deterioration of heart failure and protecting heart function. MitoQ is another mitochondria-targeted ROS scavenger. Studies have shown that the application of MitoQ can specifically reduce hypoxic pulmonary vasoconstriction, but it does not inhibit pulmonary vascular remodeling and the development of chronic hypoxic-induced pulmonary hypertension, and at the same time weaken right ventricular dilation [58]. In this experiment, similar therapeutic effects were achieved with the intervention of SKQ1. However, the long-term use of antioxidants or anti-inflammatory strategies as pharmacological treatments does not have significant efficacy in cardiovascular disease [26] but rather weakens the body’s own antioxidant capacity and makes patients drug-dependent.

In contrast to antioxidants, aerobic exercise as a non-pharmacological measure to prevent or treat a variety of cardiovascular diseases protects heart function through a variety of mechanisms. Regular exercise has a positive effect on cardiopulmonary function and can promote the antioxidant capacity of patients with grade II and III heart failure [59]. Activation of AMPK is considered a key antioxidant mechanism [60], which promotes AMPK expression, increases G6PD enzyme activity, and enhances the antioxidant capacity of nonenzymatic GSH [61]. AICAR, an AMPK agonist, counteracts palmitate-induced increase in mitochondrial ROS and inhibits inflammation and apoptosis [62,63]. Similar results were found in this study. AICAR can alleviate ROS induced by AngⅡ, increase mitochondrial membrane potential and inhibit mitochondrial fragmentation in primary cardiomyocytes. Lipid peroxidation can lead to the escape of cardiolipin-linked Cytc c, increase the release of Caspase-3, and then lead to apoptosis [64]. 4-HNE is the product of lipid peroxidation under oxidative stress, and the expression of 4-HNE protein is significantly increased in heart failure induced by myocardial infarction, which promotes the activation of p53 and leads to apoptosis of the cardiomyocytes [65,66]. In this study, we found that aerobic exercise reduced the expression of 4-HNE and p53 and decreased apoptosis induced by this pathway. Mitochondrial ROS levels were elevated when ALCAT1 levels were elevated [67], while exercise training reduced the expression of ALCAT1 after myocardial infarction, increased antioxidant levels, and reduced the production of lipid peroxidation products to protect the heart from MI-induced myocardial damage [68,69,70]. In addition, previous studies of this research team have found that regular exercise can up-regulate M2AChR, activate parasympathetic nerve state [46], reduce mitochondrial ROS, enhance endothelial nitric oxide production, inhibit myocardial mitochondrial autophagy and endoplasmic reticulum stress, and reduce myocardial apoptosis, thereby resisting ischemia–reperfusion-induced myocardial injury, which improves cardiac function [71,72]. In particular, moderate–low intensity exercise induces the activation of transcription factors and promotes molecular cascades leading to increased activation of antioxidant enzymes, DNA repair enzymes, and the ubiquitin–proteasome system [73], thereby reducing the level of ROS produced, improving cell adaptation to subsequent stress, and reducing lipid peroxidation. Indirect antioxidant protection is provided by enhancing the activity of endogenous antioxidant enzymes [9,74]. Consistent with these results, we found that aerobic exercise significantly reduced myocardial oxidative stress, manifested by a decrease in DHE-positive particles, inflammation, and apoptosis. The comparison found that aerobic exercise and SKQ1 intervention had similar effects on reducing oxidative stress. Therefore, we speculated that aerobic exercise may play a beneficial role in ameliorating heart failure by activating endogenous antioxidant mechanisms and inhibiting mitochondrial ROS.

## 4. Materials and Methods

### 4.1. Experimental Animals and Groups

Sixty 9-week-old C57BL/6 male mice were ordered from Beijing Weitonglihua Laboratory Animal Technology Co., Ltd. (Beijing, China). Animal license number: SCXK (Beijing) 2021-0006, as well as separate cages, temperature (22 ± 2) °C, and free drinking water. The animal study was approved by the Ethics Committee of Shaanxi Normal University. The mice were randomly divided into 4 groups: the SHAM operation group (SHAM), heart failure group (HF), heart failure + aerobic exercise group (HE), and heart failure + SKQ1 intervention group (HS). The model adopted transverse aortic constriction (TAC) to establish the heart failure induced by pressure overload [75], and only the SHAM group underwent a thoracotomy without an aortic arch ligation.

### 4.2. Exercise and SKQ1 Intervention Program

The mice were left to rest for one week after surgery. In the HE group, reference was made to the study of Julia Bottner et al. [76,77]; 8–9 m/min was served in the warm-up stage for 5 min for 1 h every day. After 50 min, 12–13 m/min, relaxation stage 5 min, 8–9 m/min, 6 days a week, with continuous training for 9 weeks. In the HS group, according to the study of Demyanenko et al. [78,79], one week after the operation, SKQ1 was added to the drinking water at a dose of 100 nmol/kg/d for 9 weeks.

### 4.3. Cardiac Function Tests

An ultrasonic tachycardia recorder (VINNO6VET, VINNO) was collected for the detection and analysis of echocardiograms. After isoflurane anesthesia, the ultrasound probe was measured along the long axis of the mouse heart. In M mode, the mean values of at least three consecutive cardiac cycles were recorded, and measures of EF, FS, LVIDs, and LVIDd were taken to assess cardiac function.

### 4.4. Morphological Studies and Analyses

The changes in myocardial microstructure and ultrastructure were observed by histological staining and transmission electron microscopy. The heart was fixed in 4% paraformaldehyde, and after rinsing and dehydration, the heart was longitudinally or transversally dipped in wax, and the section thickness was 5 μm. H&E and WGA (20 μg/mL) staining were used to observe the size of the cardiomyocytes [80]. Masson’s staining assessed myocardial fibrosis. TUNEL staining was used to evaluate myocardial apoptosis. Myocardial oxidative stress was assessed by DHE staining. The morphological and structural changes in the myocardial tissue and mitochondria were observed by transmission electron microscopy.

### 4.5. Western Blotting

After sampling, 50~60 mg of myocardial tissue was taken from the left ventricle, and protein was extracted and quantified. After electrophoresis, membrane transfer and closure occurred. Then, the membrane and the primary antibody were gently shaken at 4 degrees and incubated overnight. The primary antibody was diluted and incubated as follows: MFN2 (1:100,000, 9482S, Cell Signaling Technology, Inc. (CST), Boston, MA, USA); OPA1 (1:100,000, 67589S, CST); DRP1 (1:100, 8570S, CST); PINK1 (1:200, ab23707, abcam, Abcam plc, Cambridge, UK); BAK (1:100,000, 12105, CST); BNIP3 (1:100,000, 13795, CST); Parkin (1:600, sc-32282, Santacruz, Santa Cruz Biotechnology, Inc., Santa Cruz, CA, USA); NLRP3 (1:1000, 15101S, CST); Caspase-1 (1:100,000, 89332S, CST); IL-1β (1:100,000, 12242S, CST); BAX (1:100,000, 41162, CST); and HIF-1α (1:100,000, 36169, CST). The corresponding secondary antibody (1:8000, Tiande Yue, Beijing TDY Biotech Co., Ltd., Beijing, China) was incubated at room temperature for 1 h. ECL luminescent solution was added to the membrane for gel imaging.

### 4.6. Primary Cardiomyocyte Isolation and Culture

Primary neonatal rat cardiomyocytes were prepared as follows [81]. The hearts of 1–3-day-old neonatal rats were digested with 0.08% type II collagens, 0.04% trypsin, and D-Hank’s solution. Cardiomyocytes were collected by centrifuge, then inoculated in a 6 cm Petri dish after being suspended and filtered. The cells were grown in humid air containing 5% CO_2_ at 37 °C. After 48 h, we changed the liquid, and then changed the liquid the next day. After the cell density reached 80–90%, they were randomly divided into 4 groups: the control group (C), AngⅡ group (A), AngⅡ + SKQ1 group (A + S), and AngⅡ + AICAR group (A + A), and treated with culture medium containing AngⅡ (1 μM), SKQ1 (0.5 mM), and AICAR (1 mM) for 24 h, respectively.

### 4.7. MitoTracker Red and JC-1 Staining

After the cardiomyocyte intervention was completed, 1 mL of JC-1 staining working solution, DHE staining working solution, or MitoTracker Red staining working solution was prepared from MEM culture fluid. The culture solution was aspirated and washed once with PBS (sterile) to aspirate the residual liquid. Afterwards, the environment was protected from light, JC-1 (37 °C, 30 min), DHE (37 °C, 30 min), MitoTracker Red (37 °C, 15 min). At the end of incubation, the working solution was aspirated and washed twice with PBS. Then, 1 mL of culture solution was added, followed by observation with a fluorescence microscope or laser confocal microscope.

### 4.8. Data Analysis and Processing

Unless otherwise stated, results show the mean ± standard deviation of at least three independent experiments. Differences between groups were analyzed by one-way ANOVA or independent *t*-tests. Values of *p* < 0.01 (**) and *p* < 0.05 (*) were considered statistically significant, and all data were plotted using GraphPad Prism 8.0.

## 5. Conclusions

**Figure 8:**
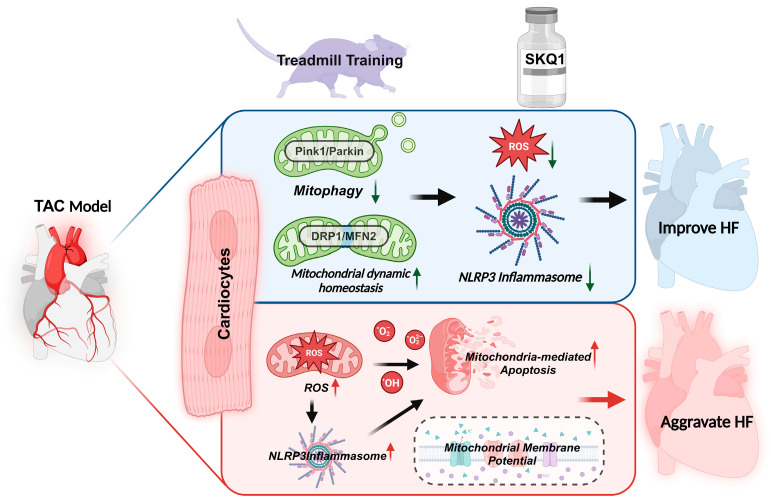
Molecular mechanisms of aerobic exercise and SKQ1 inhibition of mitochondrial ROS to improve heart ailure.

In summary, this study demonstrated that aerobic exercise inhibited TAC-induced mitochondrial ROS production, regulated mitochondrial quality control, reduced mitochondrial damage, and alleviated myocardial oxidative stress in mice with myocardial failure. At the same time, TXNIP-mediated NLRP3 activation was decreased, thereby alleviating TAC-induced myocardial inflammation and apoptosis in mice with heart failure. Aerobic exercise and SKQ1 intervention have similar effects on the improvement of cardiac function, which may be an important mechanism for aerobic exercise to exert endogenous antioxidant mechanism, thus inhibiting the ROS-mediated vicious cycle and improving cardiac function in heart failure (Figure 8). Specific endogenous antioxidant targets need to be further studied. This study provides a new perspective for understanding the role of aerobic exercise in improving the endogenous antioxidant mechanism of heart failure, and has important theoretical basis and experimental reference value for the prevention and treatment of heart failure.

## Data Availability

The original contributions presented in the study are included in the article; further inquiries can be directed to the corresponding author.

## Abbreviations

Ang II	Angiotensin II
BAX	BCL2-associated X protein
Bcl-2	B-cell lymphoma-2
CVF	Collagen volume fraction
Cyt c	Cytochrome c
DRP1	Mitochondrial dynamin-related protein 1
EF	Ejection fraction
FS	Fractional shortening
HF	Heart failure
IL-1β	Interleukin-1β
IL-18	Interleukin-18
LVIDd	left ventricular end-diastolic internal diameter
LVIDs	left ventricular internal diameter at end-systole
MFN2	Mitofusin 2
MFN1	Mitofusin 1
MDA	Malondialdehyde
MtDNA	Mitochondrial DNA
NF-κB	Nuclear factor-kappa B
ROS	Reactive oxygen species
OPA1	Optic Atrophy 1
PINK1	PTEN induced putative kinase 1
TAC	Transverse aortic constriction
TGF-β	Transforming growth factor-β
TXNIP	Thioredoxin-interacting protein
